# Synthetic and real-world datasets for crosswalk segmentation under diverse weather and lighting conditions

**DOI:** 10.1016/j.dib.2025.111755

**Published:** 2025-06-07

**Authors:** Krešimir Romić, Hrvoje Leventić, Marija Habijan, Irena Galić

**Affiliations:** Faculty of Electrical Engineering, Computer Science and Information Technology Osijek, Kneza Trpimira 2B, Osijek HR-31000, Croatia

**Keywords:** Adverse conditions simulation, Crosswalk segmentation, First-person view, Stable diffusion, Visually impaired

## Abstract

This article presents a new dataset for crosswalk segmentation targeting assistive technologies for visually impaired individuals. The dataset combines synthetic and real-world first-person view images with corresponding binary segmentation masks. The synthetic portion contains 3000 images generated using a fine-tuned Stable Diffusion model, with 1500 images created using a standard prompt ("a crosswalk image") and 1500 additional images incorporating various environmental conditions (sunny, cloudy, rainy, and night) through specialized prompts. The real-world component comprises 300 images extracted from chest-mounted smartphone video recordings of pedestrians approaching crosswalks, carefully distributed across different environmental conditions (120 sunny, 60 cloudy, 60 rainy, and 60 night images). To ensure diversity, each physical crosswalk location appears in at most two images from different approach directions. All images in both synthetic and real-world sets were manually annotated using a custom interface where annotators defined crosswalk regions as quadrilateral polygons, creating binary masks. The dataset is organized hierarchically by image source (synthetic/real-world) and environmental condition, with consistent subfolder structures for images and their corresponding masks. This dataset addresses the scarcity of publicly available crosswalk segmentation data with environmental diversity and has potential applications in developing and benchmarking computer vision algorithms for assistive navigation systems, investigating synthetic data augmentation efficacy, and advancing pedestrian safety technologies.

Specifications Table

**Table:** 

Subject	Computer Sciences
Specific subject area	First-person view crosswalk segmentation
Type of data	Image (.jpg)
Data collection	The synthetic dataset was created using Python programming language and fine-tuned stable diffusion model for text-to-image generation. The images for real-world dataset were extracted from video sequences captured while person wearing chest-mounted camera was approaching the crosswalk. Both datasets are balanced to include adequate proportions of various environmental conditions (sunny, cloudy, rainy, and nighttime). In synthetic dataset, conditions were simulated artificially, while in real-world dataset, data was captured during particular conditions.
Data source location	Faculty of Electrical Engineering, Computer Science and Information Technology Osijek, Kneza Trpimira 2B, Osijek, HR-31000, Croatia
Data accessibility	Repository name: FPVCrosswalk2025: A dataset for first-person view crosswalk segmentation in adverse weather and lighting conditions Data identification number: 10.17632/mcr2jwk5bp.1 Direct URL to data: https://data.mendeley.com/datasets/mcr2jwk5bp/1
Related research article	

## 1 Value of the Data

• This dataset provides a unique combination of synthetic and real-world crosswalk images with binary segmentation masks, addressing the scarcity of publicly available labeled crosswalk segmentation datasets.
• The synthetic portion (3000 images) showcases various weather and lighting conditions, creating a diverse training resource for machine learning models.
• The real-world portion (300 images) offers authentic pedestrian-perspective crosswalk images, valuable for testing and validation in real-world applications.
• Researchers can use this dataset to develop and benchmark computer vision algorithms for crosswalk detection and segmentation in assistive technologies.
• The dataset enables investigation into the efficacy of synthetic data augmentation for improving model performance in safety-critical applications.
• It specifically addresses the needs of visually impaired assistance systems, potentially contributing to improved navigation safety and independence.

## 2 Background

The development of assistive technologies for visually impaired individuals presents significant challenges, particularly in creating reliable navigation systems for urban environments. Pedestrian crosswalks represent critical safety infrastructure that must be accurately detected to ensure safe mobility for those with visual impairments [1]. Current computer vision approaches for crosswalk detection and segmentation require extensive training data to achieve robust performance across diverse real-world scenarios [2]. However, publicly available datasets specifically focused on first-person view crosswalk segmentation remain scarce. While datasets like CDSet [3] and X-CDNet [4] offer valuable crosswalk data, they are primarily captured from a vehicle perspective, targeting autonomous driving applications. Other datasets, such as UrOAC [5], provide pedestrian-view images but often utilize bounding box annotations and lack fine-grained polygonal segmentation masks critical for developing robust assistive navigation systems for visually impaired individuals. Existing collections often lack variety in environmental conditions such as different weather and lighting variations, all of which significantly impact the visual characteristics of crosswalks. These limitations can result in models that perform inadequately in real-world deployment scenarios. Our work aims to fill this specific gap by providing a first-person view dataset with detailed segmentation masks and diverse, structured environmental conditions. The emergence of text-to-image diffusion models offers a promising approach to dataset augmentation, potentially addressing the data scarcity problem by generating synthetic training images with precisely controlled environmental variations. This dataset was compiled to explore this potential by providing both synthetic crosswalk images with systematically varied conditions and a complementary real-world validation set, thereby creating a resource for advancing computer vision capabilities in assistive technologies for the visually impaired.

## 3 Data Description

**Fig. 1:**
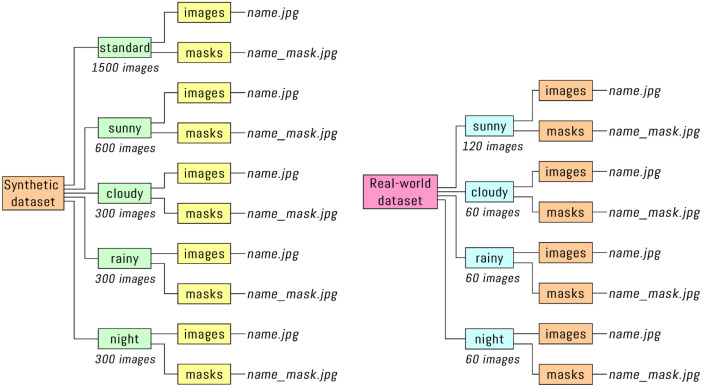
Folder structure for synthetic and real-world dataset.

The dataset [6] is organized into two main directories: “Synthetic dataset” and “Real-world dataset”, each containing crosswalk images and their corresponding binary segmentation masks arranged in a structured hierarchy based on environmental conditions as shown in Fig. 1.

Both datasets follow a consistent organizational and format pattern:

• Each environmental condition has its own dedicated folder
• Every condition folder contains two subfolders: **images** (containing the crosswalk images) and **masks** (containing the corresponding binary segmentation masks)
• All images and masks have the resolution of 640×360 pixels and are saved in .jpg format
• File naming convention: Each image file (name.jpg) has a corresponding mask file with identical naming plus the "_mask" suffix (name_mask.jpg)

The synthetic dataset contains 3000 artificially generated crosswalk images created through a fine-tuned Stable Diffusion model [7], organized into the following subfolders:

• **standard**: 1500 images generated with the basic prompt “a crosswalk image”
• **sunny**: 600 images specifically generated with prompt “a crosswalk image, sunny weather”
• **cloudy**: 300 images specifically generated with prompt “a crosswalk image, cloudy weather”
• **rainy**: 300 images specifically generated with prompt “a crosswalk image, rainy weather”
• **night**: 300 images specifically generated with prompt “a crosswalk image, night conditions”

The real-world dataset consists of 300 actual crosswalk images extracted from smartphone video recordings, distributed across the following condition folders:

• **sunny**: 120 real-world images captured during sunny weather conditions
• **cloudy**: 60 real-world images captured during cloudy weather conditions
• **rainy**: 60 real-world images captured during rainy weather conditions
• **night**: 60 real-world images captured during nighttime lighting conditions

Additionally, the dataset includes a “Fine-tuning seed images” folder containing the 150 original crosswalk images that were used to fine-tune the diffusion model for synthetic data generation.

## 4 Experimental Design, Materials and Methods

The synthetic portion of the dataset was created through a fine-tuned text-to-image diffusion model approach. Initially, 150 first-person view (FPV) images of crosswalks were collected and used to fine-tune the Stable Diffusion model, which are included in the “Fine-tuning seed images” folder to enable reproducibility and transparency of the synthetic data generation process. This fine-tuning process adapted the general-purpose image generation capabilities of Stable Diffusion to focus specifically on realistic crosswalk scenes from a pedestrian's perspective.

**Fig. 2:**
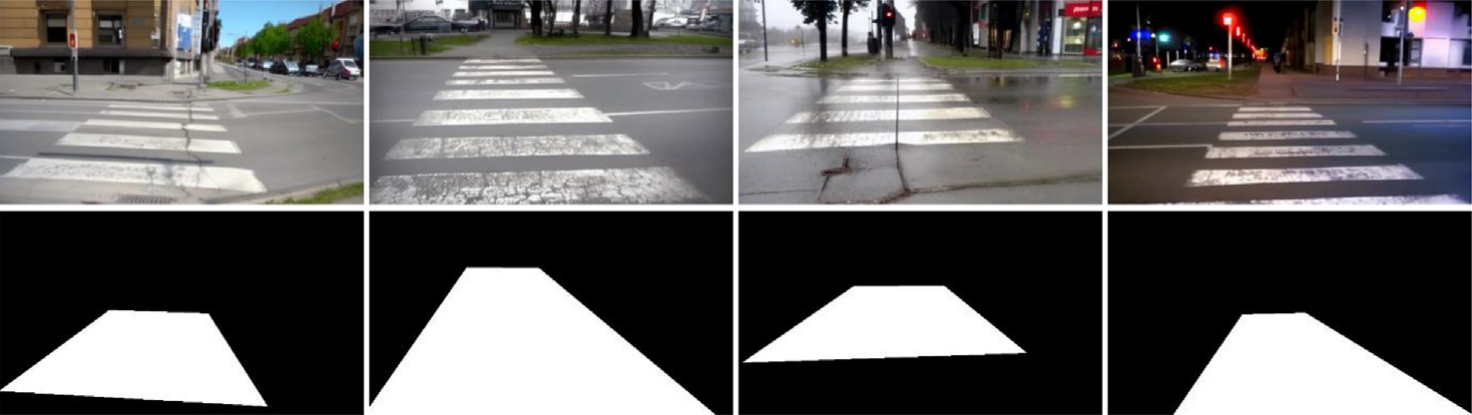
Examples of synthetically generated crosswalk images (top) and corresponding binary segmentation masks (bottom).

Following the fine-tuning process, synthetic images were generated in two distinct sets:

1. The first set of 1500 images was created using a base prompt of "a crosswalk image." These images represent crosswalks under relatively neutral environmental conditions.
2. The second set of 1500 images was generated using the base prompt with additional environmental condition modifiers [8]. These prompts followed the pattern "a crosswalk image, [condition]" where the condition included specification of weather (sunny, cloudy, rainy) or time of day (night). This approach produced 600 images with sunny conditions, 300 with cloudy conditions, 300 with rainy conditions, and 300 with nighttime conditions. Examples of synthetically generated crosswalk images are given in Fig. 2.

The two sets (standard and condition-specific) were intentionally kept separate in the dataset organization to provide flexibility in their usage. Researchers may choose to use only the standard set for general-purpose applications, only the condition-specific set for specialized scenarios, or combine both sets depending on their research requirements.

**Fig. 3:**
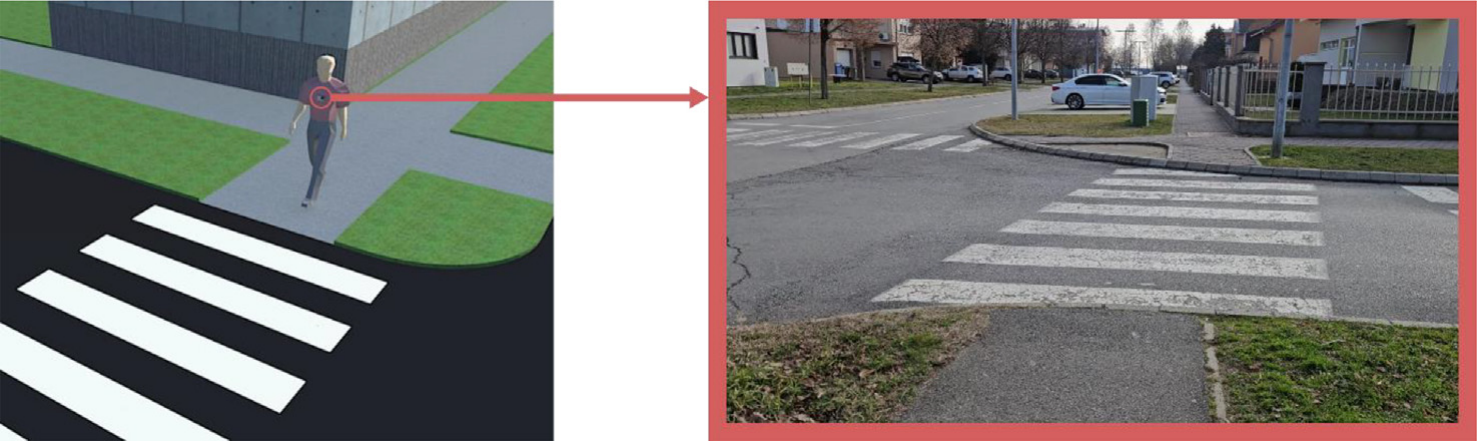
Capturing crosswalk images in first-person view (FPV).

The real-world portion of the dataset was acquired through video recording in authentic urban environments using Samsung Galaxy S23 smartphone camera (50MP main sensor, f/1.8 aperture). A smartphone was mounted at chest height on a pedestrian to capture a natural first-person perspective while approaching crosswalks (Fig. 3.). This chest-level mounting position was deliberately chosen over a head-mounted position to enhance image stability and minimize motion artifacts (e.g., blur and excessive jitter) often associated with natural head movements during walking. Our preliminary tests indicated that this setup yielded more consistent video frames, which is beneficial for precise manual annotation and effective model training, while still providing a natural first-person perspective crucial for pedestrian navigation. Moreover, this placement reflects the common positioning of many wearable assistive devices designed for visually impaired individuals, thereby increasing the direct applicability of our dataset to real-world scenarios. Video sequences were recorded across four distinct environmental conditions: sunny (120 samples), cloudy (60 samples), rainy (60 samples), and nighttime (60 samples).

**Fig. 4:**
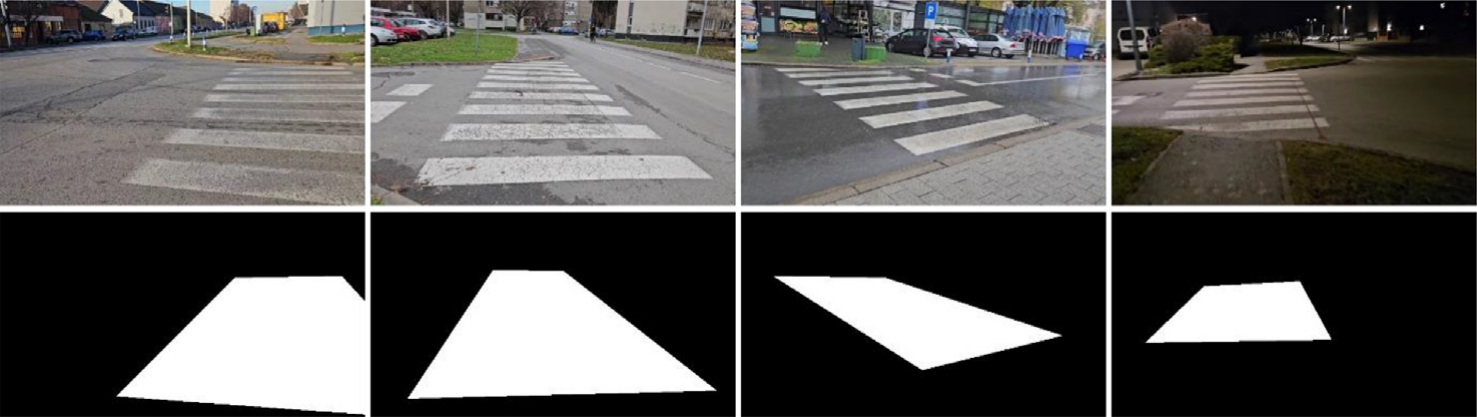
Examples of real-world crosswalk images (top) and corresponding binary segmentation masks (bottom).

Individual frames were extracted from these video sequences to form the dataset images. This approach intentionally preserved natural motion artifacts and sensor noise typical of real-world wearable camera systems [9]. To ensure diversity and prevent data redundancy, a maximum of two images (from opposite approach directions) were included from any single physical crosswalk location. This constraint enhanced the variety of crosswalk appearances, layouts, and surrounding environments within the dataset while maintaining balance across the environmental conditions. Examples of captured real-world images from dataset are shown in Fig. 4.

Both synthetic and real-world images were annotated using an identical methodology to ensure consistency. A custom annotation interface was developed that allowed sequential processing of images within each folder. For each image, a human annotator identified the crosswalk region by selecting four points to define an irregular quadrilateral polygon encompassing the crosswalk area.

The resulting binary segmentation masks use a simple two-colour scheme: white pixels (255) indicate the crosswalk region, while black pixels (0) represent the background. This annotation approach provides clear ground truth for semantic segmentation tasks while maintaining a straightforward format compatible with most computer vision frameworks.

## Limitations

Despite efforts to create a comprehensive dataset, several limitations should be acknowledged. The synthetic data, while generally realistic, occasionally contains artifacts or unrealistic elements in specific areas of some images, particularly when depicting people or vehicles in the crosswalk surroundings. These visual anomalies are an inherent limitation of current text-to-image diffusion models when generating complex urban scenes.

The dataset focuses exclusively on zebra-style crosswalks, which predominate in European countries but may limit applicability in regions where different crosswalk designs are used. This specificity constrains the dataset's utility for developing truly universal crosswalk detection systems.

Although the dataset incorporates various environmental conditions, it may not adequately represent all possible outlier scenarios like extreme weather conditions (heavy snow, fog, sandstorms) or unusual lighting situations (glare, flickering streetlights).

## Ethics Statement

The authors state that the current work meets the ethical requirements for publication in Data in Brief and confirm that the current work does not involve human subjects, animal experiments, or any data collected from social media platforms.

## CRediT Author Statement

**Krešimir Romić**: Methodology, Investigation, Data Curation, Writing - Original Draft, Visualization. **Hrvoje Leventić**: Conceptualization, Writing - Review & Editing. **Marija Habijan**: Visualization, Writing - Review & Editing. **Irena Galić**: Supervision, Writing - Review & Editing

## Data Availability

Mendeley DataFPVCrosswalk2025: A dataset for first-person view crosswalk segmentation in adverse weather and lighting conditions (Original data)
